# Ischemic preconditioning protects neurons from damage and maintains the immunoreactivity of kynurenic acid in the gerbil hippocampal CA1 region following transient cerebral ischemia

**DOI:** 10.3892/ijmm.2015.2171

**Published:** 2015-04-07

**Authors:** JAE-CHUL LEE, HYUN-JIN TAE, GEUM-SIL CHO, IN HYE KIM, JI HYEON AHN, JOON HA PARK, BAI HUI CHEN, JEONG-HWI CHO, BICH NA SHIN, JUN HWI CHO, EUN JOO BAE, JINSEU PARK, YOUNG-MYEONG KIM, SOO YOUNG CHOI, MOO-HO WON

**Affiliations:** 1 Department of Neurobiology, School of Medicine, Kangwon National University, Chuncheon 200–701, Republic of Korea; 2 Department of Biomedical Science and Research Institute for Bioscience and Biotechnology, Hallym University, Chuncheon 200–702, Republic of Korea; 3 Department of Neuroscience, College of Medicine, Korea University, Seoul 136–705, Republic of Korea; 4 Department of Physiology, College of Medicine, and Institute of Neurodegeneration and Neuroregeneration, Hallym University, Chuncheon 200–702, Republic of Korea; 5 Department of Emergency Medicine, School of Medicine, Kangwon National University, Chuncheon 200–701, Republic of Korea; 6 Department of Pediatrics, Chuncheon Sacred Heart Hospital, College of Medicine, Hallym University, Chuncheon 200–701, Republic of Korea; 7 Department of Molecular and Cellular Biochemistry, School of Medicine, Kangwon National University, Chuncheon 200–701, Republic of Korea

**Keywords:** ischemic preconditioning, transient ischemia, CA1 pyramidal neurons, delayed neuronal death, kynurenic acid

## Abstract

Pyramidal neurons in region I of hippocampus proper (CA1) are particularly vulnerable to excitotoxic processes following transient forebrain ischemia. Kynurenic acid (KYNA) is a small molecule derived from tryptophan when this amino acid is metabolized through the kynurenine pathway. In the present study, we examined the effects of ischemic preconditioning (IPC) on the immunoreactivity and protein levels of KYNA following 5 min of transient forebrain ischemia in gerbils. The animals were randomly assigned to 4 groups (sham-operated group, ischemia-operated group, IPC + sham-operated group and IPC + ischemia-operated group). IPC was induced by subjecting the gerbils to 2 min of ischemia followed by 1 day of recovery. In the ischemia-operated group, we observed a significant loss of pyramidal neurons in the CA1 stratum pyramidale (SP) at 5 days post-ischemia; however, in the IPC + ischemia-operated group, the pyramidal neurons were well protected. KYNA immunoreactivity in the SP of the ischemia-operated group was significantly altered following ischemia-reperfusion and was very low 5 days following ischemia-reperfusion. In the IPC + ischemia-operated group, however, KYNA immunoreactivity was constitutively detected in the SP of the CA1 region after the ischemic insult. We also found that the alteration pattern of the KYNA protein level in the CA1 region following ischemia was generally similar to the immunohistochemical changes observed. In brief, our findings demonstrated that IPC maintained and even increased KYNA immunoreactivity in the SP of the CA1 region following ischemia-reperfusion. The data from the present study thus indicate that the enhancement of KYNA expression by IPC may be necessary for neuronal survival following transient ischemic injury.

## Introduction

Transient forebrain ischemia induced by the deprivation of brain blood flow leads to irreversible brain damage in specific vulnerable areas of the brain (1–3). Delayed neuronal death occurs several days after ischemia-reperfusion injury in the stratum pyramidale (SP) of region I of hippocampus proper (CA1) (4–7). One of the mechanisms of delayed neuronal death is possibly associated with a number of biochemical events triggered by glutamate excitotoxicity (8–10).

Ischemic preconditioning (IPC) represents an important adaptation of the central nervous system (CNS) to sublethal ischemia, which can increase the ischemic tolerance of the CNS to a subsequent longer or lethal period of ischemia (11,12). IPC induces the expression of diverse genes involved in cytoprotection and, in turn, encodes proteins that lead to the enhancement of resistance to cerebral ischemia (13). This phenomenon is termed cerebral ‘ischemic tolerance’, although the basic mechanisms underlying cerebral ischemic tolerance are not yet fully understood (14).

Kynurenic acid (KYNA) is an endogenous metabolite of the kynurenine pathway for tryptophan degradation and is produced from its precursor L-kynurenine (KYN) by the enzyme, kynurenine-aminotransferase (15). The formation of KYNA in particular, has been shown to play an important role in the CNS at physiological concentrations, as this metabolite selectively acts as an antagonist of N-methyl-D-aspartate (NMDA) receptors by blocking the co-agonist site for glycine (16,17), as well as a non-competitive inhibitor of α7-nicotinic receptors for acetylcholine (18). Thus, KYNA protects neuronal cells from excitotoxicity evoked by the overactivation of NMDA receptors. Furthermore, KYNA plays a versatile role in pathological states, including inflammatory (19), vascular (20) and antioxidant (21) processes. Indeed, the exogenous administration of KYNA or its enhanced endogenous synthesis has been proven to be a robust neuroprotectant in animal models of forebrain ischemia (22–25). However, research on KYNA as a neuroprotective agent is rather limited, as it hardly crosses the blood-brain barrier (22,26).

To the best of our knowledge, the expression patterns of endogenous KYNA immunoreactivity in the IPC-mediated hippocampus following transient forebrain ischemia have not been studied thus far. Therefore, the present study was carried out to investigate the temporal changes and specific roles of endogenous KYNA in the IPC-induced neuroprotective effects against transient ischemic damage in the hippocampus of gerbils, which are considered to be a good animal model for studying transient cerebral ischemia (27,28).

## Materials and methods

### Experimental animals

We used the progeny of male Mongolian gerbils (*Meriones unguiculatus*) obtained from the Experimental Animal Center, Kangwon National University, Chuncheon, Korea. The gerbils were used at 24 weeks of age (body weight, 65–75 g) and were maintained under pathogen-free conditions with a temperature of 23°C and a humidity of 60%. All the experimental protocols were approved by the Institutional Animal Care and Use Committee (IACUC) at Kangwon National University and adhered to guidelines that are in compliance with the current international laws and policies (Guide for the Care and Use of Laboratory Animals, The National Academies Press, 8th edition, 2011).

### Animal groups and induction of transient forebrain ischemia

The animals were divided into 4 groups (n=7 at each time point): i) group 1, the sham-operated group: the bilateral common carotid arteries were exposed and the animals were not subjected to ischemia (sham-operation); ii) group 2, the ischemia-operated group: the animals were subjected to 5 min of transient forebrain ischemia; iii) group 3, the IPC + sham-operated group: the animals were subjected to 2 min of sublethal ischemia prior to sham-operation; and iv) group 4, the IPC + ischemia-operated group: the animals were subjected to 2 min of sublethal ischemia prior to 5 min of transient ischemia. The IPC paradigm has been proven to be very effective at protecting neurons against ischemic damage in this ischemic model (29). The animals in groups 2 and 4 were allowed to recover for different periods of time (sham, 1 day, 2 days and 5 days), as pyramidal neurons in the hippocampal CA1 region survive for 3 days and then begin to die 4–5 days following ischemia-reperfusion.

Transient forebrain ischemia was developed according to the method described in our previous study (30). In brief, the experimental animals were anesthetized with a mixture of 2.5% isoflurane in 33% oxygen and 67% nitrous oxide. Ischemia was induced by the occlusion of arteries with non-traumatic aneurysm clips (Yasargil FE 723K; Aesculap, Tuttlingen, Germany). After 2 or 5 min of occlusion, the aneurysm clips were removed from the common carotid arteries. The body (rectal) temperature under free-regulating or normothermic (37±0.5°C) conditions was monitored with a rectal temperature probe (TR-100; Fine Science Tools, Foster City, CA, USA) and maintained using a thermometric blanket before, during and after surgery until the animals had completely recovered from the anesthesia. Thereafter, the animals were kept in a thermal incubator (temperature, 23°C; humidity, 60%) (Mirae Medical Industry, Seoul, Korea) to maintain the body temperature of the animals until they were sacrificed (as described below).

### Tissue processing for histological analysis

For histological analysis, the gerbils (n=7 at each time point) were deeply anesthetized with pentobarbital sodium and perfused through the left ventricle with 0.1 M phosphate-buffered saline (PBS, pH 7.4) followed by 4% paraformaldehyde in 0.1 M phosphate-buffer (PB, pH 7.4). The brains were removed and post-fixed in the same fixative for 6 h. The brain tissues were embedded in tissue-freezing medium and serially sectioned into 30-*μ*m coronal sections using a cryostat (Leica, Wetzlar, Germany).

### Cresyl violet (CV) staining and Fluoro-Jade B (F-J B) histofluorescence

To investigate the delayed neuronal damage in the hippocampus following ischemia-reperfusion, CV staining and F-J B histofluorescence were performed as previously described (31). In brief, the sections were stained with 1.0% (w/v) CV acetate (Sigma-Aldrich, St. Louis, MO, USA) and dehydrated. They were then mounted with Canada balsam (Kanto Chemical Co., Tokyo, Japan). For F-J B histofluorescence, the sections were immersed in a 0.0004% F-J B staining solution (Histochem Inc., Jefferson, AR, USA). After washing, the sections were examined using an epifluorescent microscope (Carl Zeiss, Göttingen, Germany) with blue (450–490 nm) excitation light and a barrier filter.

### Immunohistochemistry for neuronal nuclei (NeuN) and KYNA

For immunohistochemical staining, the sections were prepared out according to the method described in our previous study (31). The brain sections were blocked with 10% normal goat serum in 0.05 M PBS followed by staining with primary mouse anti-NeuN antibody (a neuron-specific soluble nuclear antigen) (diluted 1:1,000; MAB377; Chemicon International, Temecula, CA, USA) and rabbit anti-KYNA antibody (diluted 1:200; ab37105; Abcam, Cambridge, MA, USA) overnight at 4°C. The sections were then incubated with the secondary antibodies (BA-9200, BA-1000; Vector Laboratories Inc., Burlingame, CA, USA) and were developed using the Vectastain ABC system (Vector Laboratories Inc.). Subsequently, they were visualized with 3,3′-diaminobenzidine in 0.1 M Tris-HCl buffer. In order to establish the specificity of the immunostaining, a negative control test was carried out with pre-immune serum instead of primary antibody. The negative control resulted in the absence of immunoreactivity in any structures.

### Western blot analysis

To obtain the accurate data for changes in the protein levels of KYNA in the hippocampal CA1 region following transient forebrain ischemia abd IPC, the animals (n=7 at each time point) were sacrificed at designated time points (sham, 1, 2 and 5 days) following ischemia-reperfusion and the brain tissues were used for western blot analysis. As previously described (31), after the animals were sacrificed, the brain tissues were removed and transversely cut into sections with a thickness of 400 *μ*m using a vibratome (Leica), and the hippocampal CA1 region was dissected with a surgical blade, removing the hippocampus. The tissues were homogenized in 50 mM PBS (pH 7.4) containing 0.1 mM ethylene glycolbis(2-aminoethyl Ether)-N,N,N’,N’ tetraacetic acid (EGTA; pH 8.0), 0.2% Nonidet P-40, 10 mM ethylendiaminetetraacetic acid (EDTA; pH 8.0), 15 mM sodium pyrophosphate, 100 mM β-glycerophosphate, 50 mM NaF, 150 mM NaCl, 2 mM sodium orthovanadate, 1 mM phenylmethylsulfonyl fluoride (PMSF) and 1 mM dithiothreitol (DTT). Following centrifugation (at 12,000 rpm for 10 min), the protein level was determined in the supernatants using a Micro BCA protein assay kit with bovine serum albumin as the standard (Pierce Chemical Co., Rockford, IL, USA). Aliquots containing 20 *μ*g of total protein were boiled in loading buffer containing 150 mM Tris (pH 6.8), 3 mM DTT, 6% SDS, 0.3% bromophenol blue and 30% glycerol. Subsequently, each aliquot was loaded onto a 12.5% polyacryamide gel. Following electrophoresis, the gels were transferred onto nitrocellulose transfer membranes (Pall Corp., East Hills, NY, USA). To reduce background staining, the membranes were incubated with 5% non-fat dry milk in PBS containing 0.1% Tween-20 for 45 min, and then with rabbit anti-KYNA antibody (diluted 1:1,500; Santa Cruz Biotechnology) and peroxidase-conjugated goat anti-rabbit IgG (A0545; Sigma-Aldrich), and then subjected to enhanced chemiluminescence using an ECL kit (Pierce Chemical Co.). Loading controls were performed using antibodies against β-actin (Abcam, Inc., Cambridge, MA, USA).

### Data analysis

The brain tisue sections were selected according to anatomical landmarks corresponding to target coordinates [anteroposterior (AP) diameter from −1.4 to −1.8 mm] of the gerbil brain atlas. The number of CV-positive, NeuN-immunoreactive and F-J B-positive cells was counted in a 200×200 *μ*m^2^ area, applied approximately at the center of the CA1 in the SP. Cell counts were obtained by averaging the total cell numbers from each animal per group.

In order to quantitatively analyze KYNA immunoreactivity, we applied a method described in a previous study of ours (27). In brief, the density of immunoreactive structures was evaluated on the basis of a relative optical density (ROD), which was obtained after the transformation of the mean gray level using the following formula: ROD = log (256/mean gray level).

According to a method described in a previous study of ours (28), the results of western blot analysis were quantified using Scion Image software (Scion Corp., Frederick, MD, USA), which was used to count the ROD: a ratio of the ROD was calibrated as a percentage, with the value in the sham-operated group designated as 100%.

### Statistical analysis

All data are presented as the means ± SEM. A multiple-sample comparison was applied to examine the differences between groups and days. The differences between groups on the same day were assessed by one-way ANOVA and Tukey’s post-hoc test. For the analysis of time-dependent differences between the groups, two-way ANOVA with the Bonferroni post-hoc test were used. A p-value ≤0.05 was considered to indicate a statistically significant difference.

## Results

### IPC-mediated neuroprotection

#### CV-positive (CV^+^) cells

**Figure 1:**
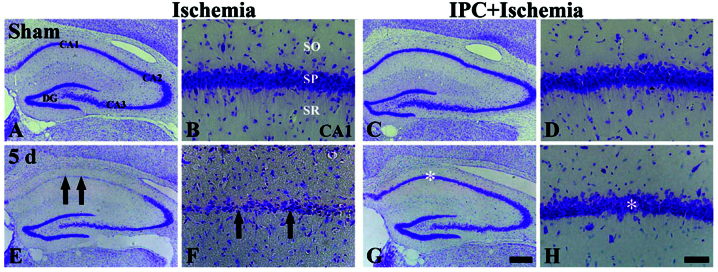
CV staining in the hippocampus of the ischemia-operated (left 2, columns) and IPC + ischemia-operated (right 2 columns) groups at (A-D) sham and (E-H) 5 days (5 d) post-ischemia. CV^+^ cells (arrows) are damaged in the stratum pyramidale (SP) of the CA1 region only at 5 days post-ischemia in the ischemia-operated group; however, CV^+^ cells (asterisk) in the IPC + ischemia-operated group were similar to those in the sham-operated group. SO, stratum oriens; SR, stratum radiatum; IPC, ischemic preconditioning; DG, dentate gyrus; CA1, CA2 and CA3 represent regions I, II and III of hippocampus proper, respectively; CV, cresyl violet. Scale bar: (A, C, E and G) 800 *μ*m and (B, D, F and H) 50 *μ*m.

We examined whether IPC is associated with a decrease in neuronal damage/death in the gerbil hippocampus following ischemia-reperfusion. CV^+^ cells were clearly observed in all the subregions of the hippocampus in the sham-operated group, and the neurons in the SP (pyramidal neurons) had a slightly large, round or pyramid-like shaped morphology (Fig. 1A and B). In the ischemia-operated group, however, 5 days after ischemia-reperfusion, the CV^+^ cells were significantly damaged in the SP of the CA1 region, but not in the CA2/3 region, compared with those of the sham-operated group (Fig. 1E); the damaged cells were shrunken and contained dark and polygonal nuclei (arrows in Fig. 1F).

In the IPC + sham-operated group, the CA1 pyramidal neurons were evidently stained with CV (Fig. 1C and D), and in the IPC + ischemia-operated group, the distribution pattern of the CV^+^ cells in the SP was very similar to that in the IPC + sham-operated group at 5 days following ischemia-reperfusion (Fig. 1G and H).

#### NeuN^+^ and F-J B^+^ cells

**Figure 2:**
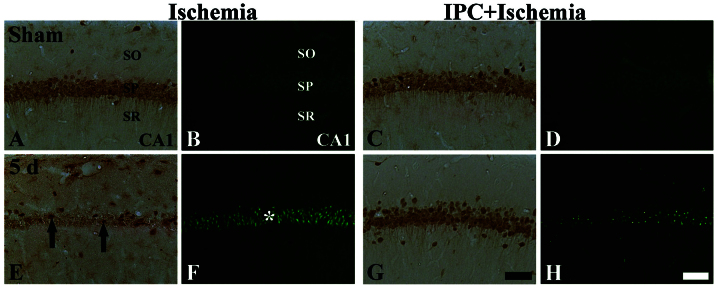
NeuN immunohistochemistry (the first and third longitudinal columns) and F-J B histofluorescence staining (the second and fourth longitudinal columns) in the CA1 region of the ischemia-operated (left 2 columns) and IPC + ischemia-operated (right 2 columns) groups at (A-D) sham and (E-F) 5 days (5 d) following ischemia-reperfusion. In the sham-operated group, many NeuN^+^ neurons, but no F-J B^+^ cells were observed in the stratum pyramidale (SP). In the ischemia-operated group, a few NeuN^+^ (black arrows) and many F-J B^+^ (asterisk) cells were detected in the SP at 5 days post-ischemia. However, in the IPC + ischemia-operated group, abundant NeuN^+^ and few F-J B^+^ cells were detected in the SP at 5 days post-ischemia. NeuN, neuronal nuclei; F-J B, Fluoro-Jade B; CA1, region I of hippocampus proper; IPC, ischemic preconditioning; SO, stratum oriens; SR, stratum radiatum. Scale bar, 50 *μ*m.

The assessment of the IPC-mediated neuroprotective effects in the CA1 region was carried out using anti-NeuN immunohistochemistry and F-J B histofluorescence staining (Fig. 2). In the sham-operated group, the pyramidal neurons in the CA1 region were evidently stained with NeuN, and no F-J B^+^ neurons were observed (Table I and Fig. 2A and B). In the ischemia-operated group, however, the number of NeuN^+^ neurons was significantly decreased in the SP of the CA1 region 5 days following ischemia-reperfusion (Table I and Fig. 2E), and, at this time point, many F-J B^+^ cells were observed in the SP of the CA1 region (Table I and Fig. 2F).

In the IPC + sham-operated group, the pyramidal neurons in the CA1 region were also evidently stained with NeuN (Fig. 2C), and no F-J B^+^ cells were observed (Table I and Fig. 2D). In the IPC + ischemia-operated group, the distribution patterns of the NeuN^+^ and F-J B^+^ cells in the SP were not significantly altered compared with those in the IPC + sham-operated group (Table I and Fig. 2G and H).

### IPC-mediated effect on KYNA immunoreactivity

#### CA1 region

**Figure 3:**
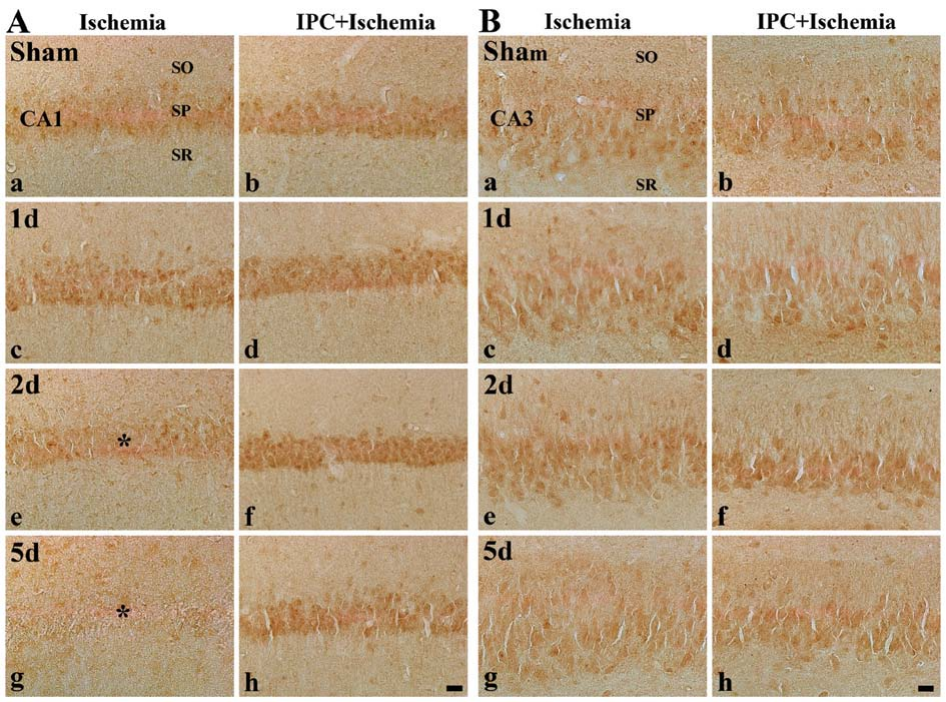
Immunohistochemistry for KYNA in (A) the CA1 region and (B) CA2/3 region of the ischemia-operated (first and third columns) and IPC + ischemia-operated (second and fourth columns) groups at (a and b) sham, (c and d) 1 day (1 d), (e and f) 2 days (2 d) and (g and h) 5 days (5 d) following ischemia-reperfusion. KYNA immunoreactivity was easily detected in the stratum pyramidale (SP) in the sham-operated group. In the CA1 region, KYNA immunoreactivity in the SP (asterisk) was increased at 1 day post-ischemia, decreased at 2 days post-ischemia and markedly decreased (reached its lowest level) at 5 days post-ischemia. However, in the IPC + sham-operated group, KYNA immunoreactivity was similar to that in the sham-operated group, and the immunoreactivity was increased in the IPC + ischemia-operated group. KYNA immunoreactivity in the SP of the CA2/3 region was not significantly altered in all groups. KYNA, kynurenic acid; CA1, region I of hippocampus propern; IPC, ischemic preconditioning; SO, stratum oriens; SR, stratum radiatum. Scale bar, 50 *μ*m.

KYNA immunoreactivity was easily detected in the SP of the CA1 region in the sham-operated group (Table II and Fig. 3A, panels a-h). In the ischemia-operated group, we found that KYNA immunoreactivity was altered in the SP following ischemia-reperfusion. The immunoreactivity was significantly increased 1 day following ischemia-reperfusion and decreased at 2 days post-ischemia (Table II and Fig. 3A, panels c and e). Five days after ischemia, KYNA immunoreactivity was even more decreased in the SP (Table II and Fig. 3A, panel g).

In the IPC + sham-operated group, KYNA immunoreactivity was similar to that in the sham-operated group (Table II and Fig. 3A, panel b). In the IPC + ischemia-operated group, KYNA immunoreactivity in the SP was a slightly increased at 1 day post-ischemia, and, thereafter, KYNA immunoreactivity in the SP was significantly higher than that in the IPC + sham-operated group (Table II and Fig. 3A, panels d, f and h).

#### CA2/3 region

In the CA2/3 region of the hippocampus of the brains of the gerbils in the sham-operated group, moderate KYNA immunoreactivity was detected in neurons of the SP (Table II and Fig. 3B, panel a). KYNA immunoreactivity was not significantly altered in the SP following ischemia-reperfusion (Table II and Fig. 3B panels c, e and g).

In the IPC + sham-operated and ischemia-operated groups, KYNA immunoreactivity in the SP in the CA1 region was similar to that in the sham-operated group (Table II and Fig. 3B panels b, d, f and h).

#### IPC-mediated effect on KYNA levels

**Figure 4:**
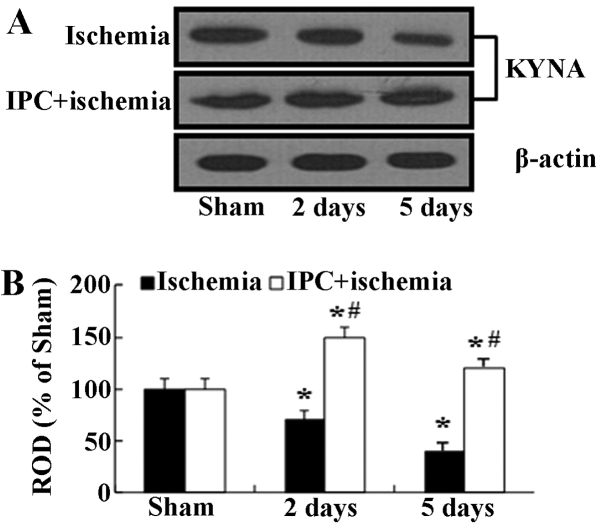
(A) Western blot analysis for kynurenic acid (KYNA) in the hippocampal CA1 region derived from the ischemia-operated and IPC + ischemia-operated groups. (B) Relative optical density (ROD) as percentage values of the western blot band is also represented (^*^P<0.05 vs. sham-operated group; #P<0.05 vs. ischemia-operated group). The bars indicate the means ± SEM.

In the present study, we examined KYNA protein levels in the hippocampal CA1 homogenates at 2 and 5 days, when the immunohistochemical data were significantly altered following ischemia-reperfusion in both groups subjected to IPC. Western blot analysis revealed that the changes in the protein expression pattern of KYNA in the CA1 region following ischemia-reperfusion were similar to those observed in the immunohistochemical data (Fig. 4). In the ischemia-operated group, the KYNA protein levels were significantly decreased at 2 days and reached their lowest levels at 5 days following ischemia-reperfusion. On the other hand, in the IPC + sham-operated group, the KYNA protein levels were slightly increased compared with the sham-operated group, and the levels in the IPC + ischemia-operated-group were similar to those in the IPC + sham-operated group (Fig. 4).

## Discussion

IPC is defined as a brief non-injurious episode of ischemia that is able to protect the brain from a subsequent longer ischemic insult (11,12). Kitagawa *et al* (32) reported the description of IPC in the brain using a gerbil model of global ischemia. IPC shows a tolerance, which has been termed ‘ischemic tolerance’ and is activated at different time points following IPC; however, the molecular mechanisms underlying ischemic tolerance are not yet fully understood (33–35).

In the present study, we chose to induce a brief period (2 min) of IPC to avoid histological tissue damage. This brief IPC stimulus did not induce neuronal damage, as assessed by CV and NeuN staining, and F-J B histofluorescence, which are sensitive markers for the detection of acute neuronal injury (11). We found that, at 5 days post-ischemia, the CA1 pyramidal neurons showed typically neuronal cell death, as shown by CV and NeuN staining, and F-J B histofluorescence. However, the viable CA1 pyramidal neurons were significantly protected from transient ischemic injury by IPC. However, although IPC often provides strong neuroprotection against ischemic brain injury, the exact mechanisms involved need to be investigated in order to develop therapeutic strategies for ischemic stroke.

KYNA, which is produced by astrocytes and neurons (18), is an endogenous metabolite of the kynurenine pathway for tryptophan degradation and is an antagonist of both NMDA and α7-nicotinic acetylcholine receptors (16–18). It is known that the level of endogenous KYNA is altered in several neurodegenerative disorders (36–38). However, data regarding the concentration of endogenous KYNA following an ischemic insult are limited. A few studies have demonstrated no change in the hippocampal content of KYNA or its decrease at 4 days after transient global ischemia (22,39,40). In the present study, we found that KYNA immunoreactivity in pyramidal neurons was evidently altered changed following ischemia and was hardly detectable in the ischemia-operated group 5 days post-ischemia.

In the present study, we found that KYNA immunoreactivity in the CA1 pyramidal neurons of the animals in the IPC + ischemia-operated-group was evidently maintained or even increased following transient ischemia. To the best of our knowledge, no studies on the effects of IPC on KYN expression have been published to date. Nevertheless, many researchers have suggested that a sufficient elevation in the KYNA content in the brain leads to a definite neuroprotective effect (41–44), although the therapeutic potential of KYNA is limited, as KYNA is hardly able to cross the blood-brain barrier (26).

Pyramidal neurons in the hippocampal CA1 region are particularly vulnerable to excitotoxic processes following transient forebrain ischemia. Excitatory amino acids (EAAs) play an important role in the pathogenesis of cerebral ischemia (45). Under ischemic conditions, the release of excess EAAs contributes to the overactivation of ionotropic NMDA receptors and α-amino-3-hydroxy-5-methyl-4-isoxazolepropionic acid (AMPA) receptors, which mediate the excessive entry of Ca^2+^, initiating glutamate-induced excitotoxicity, and finally, these excitotoxic processes eventually lead to neuronal death in the hippocampal CA1 region (46,47). Therefore, it has been shown that the blockade of the receptors for EAA (the antagonism of NMDA receptors in particular) effectively reduces neuronal damage following ischemia (48,49). Therefore, it is likely that IPC elevates the KYNA concentration sufficiently enough to affect the co-agonist site of the NMDA receptors.

In conclusion, the main findings of the present study demonstrated that in the levels of KYNA in the pyramidal neurons of the hippocampal CA1 region in animals subjected to IPC were maintained or even increased following ischemia-reperfusion and suggest that the increase in KYNA expression induced by IPC is associated with the endogenous protective response of the brain to ischemic injury.

## Figures and Tables

**Table I:** Changes in the mean number of pyramidal neurons of the hippocampal CA1 region in the ischemia-operated and IPC + ischemia-operated gerbils.

Time after IR	Ischemia-operated group		IPC + ischemia-operated group	
	NeuN^+^	F-J B^+^	NeuN^+^	F-J B+
Sham	354±14.14	0	344±12.36	0
1 day	364±16.88	0	351±15.66	0
2 days	359±13.65	5±3.72a	357±16.71	0
5 days	38±14.36^a^,^b^	141±15.77^a^,^b^	331±15.65^a^,^b^	17±6.74^a^,^b^

The mean number of NeuN^+^ and F-J B^+^ cells was counted in a 200×200 *μ*m^2^ of the stratum pyramidale of the CA1 region following ischemia-reperfusion (I-R) (n=7 per group;

a P<0.05, indicates significant difference from the corresponding ischemia-sham-operated group,

b P<0.05, indicates significant difference from the respective former group). IPC, ischemic preconditioning; IR, ischemia-reperfusion; NeuN^+^ neuronal nuclei positive; F-J B, Fluoro-Jade B.

**Table II:** Semi-quantification of the immunoreactivity of KYNA in pyramidal cells in the hippocampal CA1 and CA2/3 regions in the ischemia-operated and IPC + ischemia-operated groups.

Antibody	Region	Groups	Category	Time after ischemia-reperfusion			
				Sham	1 day	2 days	5 days
KYNA	CA1	Ischemia	CSP	+	++	±	±
		IPC + ischemia	CSP	+	++	++	++
	CA3	Ischemia	CSP	+	+	+	+
		IPC + ischemia	CSP	+	+	+	+

Immunoreactivity was scored as −, ±, + or ++, representing no staining, weakly positive, moderate or strong, respectively. CSP, cells in the stratum pyramidale; IPC, ischemic preconditioning; KYNA, kynurenic acid.

## References

[1] Kirino T (1982). Delayed neuronal death in the gerbil hippocampus following ischemia. Brain Res.

[2] Lin CS, Polsky K, Nadler JV, Crain BJ (1990). Selective neocortical and thalamic cell death in the gerbil after transient ischemia. Neuroscience.

[3] Schmidt-Kastner R, Freund TF (1991). Selective vulnerability of the hippocampus in brain ischemia. Neuroscience.

[4] Abe K, Aoki M, Kawagoe J (1995). Ischemic delayed neuronal death. A mitochondrial hypothesis. Stroke.

[5] Imon H, Mitani A, Andou Y, Arai T, Kataoka K (1991). Delayed neuronal death is induced without postischemic hyperexcitability: continuous multiple-unit recording from ischemic CA1 neurons. J Cereb Blood Flow Metab.

[6] Shelat PB, Coulibaly AP, Wang Q, Sun AY, Sun GY, Simonyi A (2006). Ischemia-induced increase in RGS7 mRNA expression in gerbil hippocampus. Neurosci Lett.

[7] Vollenweider F, Bendfeldt K, Maetzler W, Otten U, Nitsch C (2006). GABA(B) receptor expression and cellular localization in gerbil hippocampus after transient global ischemia. Neurosci Lett.

[8] Hou ST, MacManus JP (2002). Molecular mechanisms of cerebral ischemia-induced neuronal death. Int Rev Cytol.

[9] Ientile R, Caccamo D, Marciano MC (2004). Transglutaminase activity and transglutaminase mRNA transcripts in gerbil brain ischemia. Neurosci Lett.

[10] Yu S, Cai J (2003). Effects of aniracetam on extracellular levels of transmitter amino acids in the hippocampus of the conscious gerbils: an intracranial microdialysis study. Neurosci Lett.

[11] Schmued LC, Hopkins KJ (2000). Fluoro-Jade B: a high affinity fluorescent marker for the localization of neuronal degeneration. Brain Res.

[12] Lehotsky J, Burda J, Danielisova V, Gottlieb M, Kaplan P, Saniova B (2009). Ischemic tolerance: the mechanisms of neuroprotective strategy. Anat Rec (Hoboken).

[13] Gidday JM (2006). Cerebral preconditioning and ischaemic tolerance. Nat Rev Neurosci.

[14] Kardesoglu E, Isilak Z, Uz O, Yiginer O (2011). Ischemic conditioning: a current concept in reducing reperfusion injury. Chin Med J (Engl).

[15] Swartz KJ, During MJ, Freese A, Beal MF (1990). Cerebral synthesis and release of kynurenic acid: an endogenous antagonist of excitatory amino acid receptors. J Neurosci.

[16] Kemp JA, Foster AC, Leeson PD (1988). 7-Chlorokynurenic acid is a selective antagonist at the glycine modulatory site of the N-methyl-D-aspartate receptor complex. Proc Natl Acad Sci USA.

[17] Kessler M, Terramani T, Lynch G, Baudry M (1989). A glycine site associated with N-methyl-D-aspartic acid receptors: characterization and identification of a new class of antagonists. J Neurochem.

[18] Hilmas C, Pereira EF, Alkondon M, Rassoulpour A, Schwarcz R, Albuquerque EX (2001). The brain metabolite kynurenic acid inhibits alpha7 nicotinic receptor activity and increases non-alpha7 nicotinic receptor expression: physiopathological implications. J Neurosci.

[19] Moroni F, Cozzi A, Sili M, Mannaioni G (2012). Kynurenic acid: a metabolite with multiple actions and multiple targets in brain and periphery. J Neural Transm.

[20] Sas K, Csete K, Vecsei L, Papp JG (2003). Effect of systemic administration of L-kynurenine on corticocerebral blood flow under normal and ischemic conditions of the brain in conscious rabbits. J Cardiovasc Pharmacol.

[21] Lugo-Huitron R, Blanco-Ayala T, Ugalde-Muniz P (2011). On the antioxidant properties of kynurenic acid: free radical scavenging activity and inhibition of oxidative stress. Neurotoxicol Teratol.

[22] Salvati P, Ukmar G, Dho L (1999). Brain concentrations of kynurenic acid after a systemic neuroprotective dose in the gerbil model of global ischemia. Prog Neuropsychopharmacol Biol Psychiatry.

[23] Cozzi A, Carpenedo R, Moroni F (1999). Kynurenine hydroxylase inhibitors reduce ischemic brain damage: studies with (m-nitrobenzoyl)-alanine (mNBA) and 3,4-dimethoxy-[-N-4-(nitrophenyl)thiazol-2yl]-benzenesulfonamide (Ro 61-8048) in models of focal or global brain ischemia. J Cereb Blood Flow Metab.

[24] Abo M, Yamauchi H, Suzuki M, Sakuma M, Urashima M (2006). Facilitated beam-walking recovery during acute phase by kynurenic acid treatment in a rat model of photochemically induced thrombosis causing focal cerebral ischemia. Neurosignals.

[25] Germano IM, Pitts LH, Meldrum BS, Bartkowski HM, Simon RP (1987). Kynurenate inhibition of cell excitation decreases stroke size and deficits. Ann Neurol.

[26] Fukui S, Schwarcz R, Rapoport SI, Takada Y, Smith QR (1991). Blood-brain barrier transport of kynurenines: implications for brain synthesis and metabolism. J Neurochem.

[27] Lee CH, Park JH, Cho JH (2014). Changes and expressions of Redd1 in neurons and glial cells in the gerbil hippocampus proper following transient global cerebral ischemia. J Neurol Sci.

[28] Lee CH, Park JH, Choi JH, Yoo KY, Ryu PD, Won MH (2011). Heat shock protein 90 and its cochaperone, p23, are markedly increased in the aged gerbil hippocampus. Exp Gerontol.

[29] Nakamura H, Katsumata T, Nishiyama Y, Otori T, Katsura K, Katayama Y (2006). Effect of ischemic preconditioning on cerebral blood flow after subsequent lethal ischemia in gerbils. Life Sci.

[30] Lee CH, Park JH, Yoo KY (2011). Pre- and post-treatments with escitalopram protect against experimental ischemic neuronal damage via regulation of BDNF expression and oxidative stress. Exp Neurol.

[31] Lee JC, Kim IH, Cho GS (2014). Ischemic preconditioning-induced neuroprotection against transient cerebral ischemic damage via attenuating ubiquitin aggregation. J Neurol Sci.

[32] Kitagawa K, Matsumoto M, Kuwabara K (1991). ’Ischemic tolerance’ phenomenon detected in various brain regions. Brain Res.

[33] Dhodda VK, Sailor KA, Bowen KK, Vemuganti R (2004). Putative endogenous mediators of preconditioning-induced ischemic tolerance in rat brain identified by genomic and proteomic analysis. J Neurochem.

[34] Stenzel-Poore MP, Stevens SL, King JS, Simon RP (2007). Preconditioning reprograms the response to ischemic injury and primes the emergence of unique endogenous neuroprotective phenotypes: a speculative synthesis. Stroke.

[35] Feng Z, Davis DP, Sasik R, Patel HH, Drummond JC, Patel PM (2007). Pathway and gene ontology based analysis of gene expression in a rat model of cerebral ischemic tolerance. Brain Res.

[36] Kaminski RM, Zielinska E, Dekundy A, van Luijtelaar G, Turski W (2003). Deficit of endogenous kynurenic acid in the frontal cortex of rats with a genetic form of absence epilepsy. Pol J Pharmacol.

[37] Stone TW (2001). Kynurenines in the CNS: from endogenous obscurity to therapeutic importance. Prog Neurobiol.

[38] Zadori D, Klivenyi P, Szalardy L, Fulop F, Toldi J, Vecsei L (2012). Mitochondrial disturbances, excitotoxicity, neuroinflammation and kynurenines: novel therapeutic strategies for neurodegenerative disorders. J Neurol Sci.

[39] Saito K, Nowak TS, Markey SP, Heyes MP (1993). Mechanism of delayed increases in kynurenine pathway metabolism in damaged brain regions following transient cerebral ischemia. J Neurochem.

[40] Saito K, Nowak TS, Suyama K (1993). Kynurenine pathway enzymes in brain: responses to ischemic brain injury versus systemic immune activation. J Neurochem.

[41] Stone TW (2000). Development and therapeutic potential of kynurenic acid and kynurenine derivatives for neuroprotection. Trends Pharmacol Sci.

[42] Schwarcz R, Pellicciari R (2002). Manipulation of brain kynurenines: glial targets, neuronal effects, and clinical opportunities. J Pharmacol Exp Ther.

[43] Vamos E, Pardutz A, Klivenyi P, Toldi J, Vecsei L (2009). The role of kynurenines in disorders of the central nervous system: possibilities for neuroprotection. J Neurol Sci.

[44] Zadori D, Klivenyi P, Vamos E, Fulop F, Toldi J, Vecsei L (2009). Kynurenines in chronic neurodegenerative disorders: future therapeutic strategies. J Neural Transm.

[45] Lipton SA, Rosenberg PA (1994). Excitatory amino acids as a final common pathway for neurologic disorders. N Engl J Med.

[46] Dirnagl U, Iadecola C, Moskowitz MA (1999). Pathobiology of ischaemic stroke: an integrated view. Trends Neurosci.

[47] Endres M, Dirnagl U (2002). Ischemia and stroke. Adv Exp Med Biol.

[48] Lai TW, Zhang S, Wang YT (2014). Excitotoxicity and stroke: identifying novel targets for neuroprotection. Prog Neurobiol.

[49] Hoyte L, Barber PA, Buchan AM, Hill MD (2004). The rise and fall of NMDA antagonists for ischemic stroke. Curr Mol Med.

